# Supplementary material for: Complexome profiling of the Chlamydomonas psb28 mutant reveals TEF5 as an early PSII assembly factor
Source: Plant Cell. 2025 Mar 17;37(6):koaf055. doi: 10.1093/plcell/koaf055 (PMC12164590; doi:10.1093/plcell/koaf055)
Supplement: koaf055_Supplementary_Data [file koaf055_supplementary_data.zip › psb28_interactive.zip!/plots/intensity/qualityPlots/peptides/GYD1_Cre06.g288700.t1.2.html]

### Cre06.g288700.t1.2

Trivial name: GYD1  
SeqCoverage [median]: 47.90  
Mass: 118.87 kDa  
  
 

| Sequence | MissedCleavage | Mass [Da] | wtIntensity | muIntensity | Score |
| --- | --- | --- | --- | --- | --- |
| AAGTSLSGQAITDSVLIK | 0 | 1730.94 | 13467.65 | 39097.91 | 251.84 |
| AAQPMAIDAYPFHHDQK | 0 | 1938.90 | 7189.31 | 4583.67 | 87.57 |
| ALFDPSHTLNPGVILNR | 0 | 1863.00 | 6078.31 | 0.00 | 132.08 |
| ALNAATNHFVPVWNPYMPK | 0 | 2169.08 | 733.58 | 0.00 | 41.86 |
| ASAFIVFPDVR | 0 | 1220.66 | 7192.06 | 5125.69 | 132.39 |
| ATYNTVPEWPNK | 0 | 1418.68 | 0.00 | 420.93 | 65.35 |
| DGYSTSR | 0 | 784.34 | 927.77 | 0.00 | 95.35 |
| DITLTPR | 0 | 814.45 | 4076.16 | 5074.08 | 104.11 |
| FALYEPVEFIR | 0 | 1382.72 | 3681.51 | 3647.49 | 131.62 |
| FLKPSPAASPIVNR | 1 | 1495.85 | 12195.06 | 28039.69 | 106.32 |
| FLNIVNAMHSVVGSAPLSAISR | 0 | 2282.22 | 1918.54 | 1781.84 | 90.22 |
| FPELTGASLQHLNLPK | 0 | 1763.96 | 0.00 | 2941.61 | 115.82 |
| GAAPLKVPAPPAPAAAEASGIPR | 1 | 2108.17 | 17225.52 | 51332.42 | 148.89 |
| GAELEAALLK | 0 | 1013.58 | 14565.84 | 22113.01 | 141.52 |
| GFKDAAASK | 1 | 893.46 | 1250.71 | 2305.56 | 110.76 |
| GLIPIVGAAR | 0 | 965.60 | 4102.82 | 9550.74 | 121.52 |
| GLVYLVDEATAPK | 0 | 1374.74 | 12446.35 | 12556.63 | 161.17 |
| GLVYLVDEATAPKK | 1 | 1502.83 | 1291.51 | 1111.92 | 57.43 |
| GMRFPELTGASLQHLNLPK | 1 | 2108.12 | 2250.02 | 7068.68 | 74.43 |
| GQDEAALQSR | 0 | 1073.51 | 1650.34 | 1516.61 | 163.38 |
| HFLVDKLEWK | 1 | 1313.71 | 0.00 | 1215.84 | 53.33 |
| HLIIGSEGTLGFVSR | 0 | 1584.86 | 5180.11 | 15063.59 | 111.83 |
| ILAAHQK | 0 | 779.47 | 3528.98 | 6102.81 | 138.53 |
| INTGDLIK | 0 | 872.50 | 1152.87 | 6798.32 | 111.95 |
| KMGIEESFAK | 1 | 1138.57 | 866.88 | 0.00 | 55.81 |
| KNKLPIQYK | 2 | 1130.68 | 0.00 | 637.87 | 61.69 |
| LKQLGPGASEEEKK | 2 | 1512.81 | 2772.47 | 3831.78 | 122.20 |
| LQVPITFR | 0 | 972.58 | 6042.96 | 37648.59 | 99.00 |
| LVVKVHNEDEVRR | 2 | 1591.88 | 0.00 | 808.81 | 65.56 |
| MGIEESFAK | 0 | 1010.47 | 5050.36 | 8648.28 | 125.31 |
| MMGPAASDTETAAVHEK | 0 | 1744.78 | 18037.21 | 24027.55 | 230.96 |
| NETSVDAVELFDR | 0 | 1493.70 | 11137.18 | 17934.55 | 222.20 |
| NFTVHGDGSVITVEPGLIGGEVNR | 0 | 2466.25 | 6603.13 | 16800.39 | 118.37 |
| QLGPGASEEEKK | 1 | 1271.64 | 2309.00 | 4683.99 | 109.96 |
| QYSDPVR | 0 | 863.41 | 909.46 | 0.00 | 73.62 |
| RVQADKELTALIR | 2 | 1511.88 | 2187.50 | 3329.82 | 68.81 |
| SLVDGVVSLAR | 0 | 1114.63 | 20393.67 | 55814.83 | 170.47 |
| SQISEPSLR | 0 | 1015.53 | 14754.57 | 26057.75 | 128.12 |
| TFAYGTDASFYR | 0 | 1397.63 | 0.00 | 3961.39 | 106.40 |
| VDLVGAVSSVSESDKVEPGVFK | 1 | 2247.16 | 15267.82 | 23389.47 | 109.75 |
| VHNEDEVRR | 1 | 1152.56 | 7453.44 | 10356.57 | 116.52 |
| VLTAAGLPFGAK | 0 | 1143.67 | 23799.45 | 44395.17 | 159.37 |
| VMSLFGK | 0 | 780.42 | 1746.17 | 3383.31 | 123.99 |
| VQADKELTALIR | 1 | 1355.78 | 4480.77 | 13970.60 | 109.84 |
| YAAFVEEITK | 0 | 1169.60 | 10383.47 | 34633.23 | 120.15 |
|  |  |  | 276300.5 | 561763.0 |  |
